# Distinguishing Early Depression from Negative Emotion via Multi-Domain EEG Feature Fusion and Multi-Head Additive Attention Network

**DOI:** 10.3390/e28020218

**Published:** 2026-02-13

**Authors:** Ruoyu Du, Benbao Wang, Haipeng Gao, Tingting Xu, Shanjing Ju, Xin Xu, Jiangnan Xu

**Affiliations:** 1 School of Communication and Information Engineering, Nanjing University of Posts and Telecommunications, No. 66, XinMofan Road, Gulou District, Nanjing 210003, China; 2 School of Internet of Things, Nanjing University of Posts and Telecommunications, No. 66, XinMofan Road, Gulou District, Nanjing 210003, China

**Keywords:** EEG, depression recognition, negative emotion, feature fusion, attention mechanism

## Abstract

The early diagnosis of depression is often impeded by the subjectivity inherent in traditional clinical assessments. To advance objective screening, this study proposes a lightweight neural network framework designed to discriminate between pathological depressive states and non-pathological transient negative emotions using EEG signals. Diverging from conventional methods that rely on single-domain features, we construct a comprehensive multi-domain feature space via Wavelet Packet Decomposition. Specifically, the framework integrates frequency (*α*/*β* power spectral density ratio), spatial (normalized *α*-asymmetry), and non-linear (Sample Entropy) attributes to capture the heterogeneous neurophysiological dynamics of depression. To effectively synthesize these diverse features, a multi-head additive attention mechanism is introduced. This mechanism empowers the model to adaptively recalibrate feature weights, thereby prioritizing the most discriminative patterns associated with the disorder. Experimental validation on the DEAP (negative emotion) and HUSM (major depressive disorder) datasets demonstrates that the proposed method achieves a classification accuracy of 92.2% and an F1-score of 93%. Comparative results indicate that our model significantly outperforms baseline SVM and standard deep learning approaches. Furthermore, the architecture exhibits high computational efficiency and rapid convergence, highlighting its potential as a deployable engine for real-time mental health monitoring in clinical scenarios.

## 1. Introduction

Emotion is a complex psychological state encompassing physiological, behavioral, cognitive, and subjective dimensions, playing a crucial role in human cognition and daily interaction. Depression, a serious mental disorder affecting a significant portion of the global population, poses severe health risks and social burdens [1]. One of the most prominent features of early-stage depression is persistent low mood. With the accelerating pace of modern life, individuals face increasing psychological pressure, leading to negative emotions such as anxiety and sadness [2,3]. If not effectively alleviated, these transient negative states may progress into clinical depression.

The interplay between negative emotions and depression is intricate. Negative mood is not only a core symptom of depression but also a significant factor in its onset and persistence [4]. Therefore, understanding and identifying specific negative emotional states can play a pivotal role in the early prevention and intervention of depression [5]. While conventional diagnosis relies on subjective self-reports or clinical interviews, there is a growing need for objective physiological indicators.

While numerous studies have demonstrated the effectiveness of EEG in emotion recognition [6,7,8], significant technical challenges remain in the objective screening of depression. At present, some studies focus on the coarse-grained binary classification, but there is a lack of in-depth research on the physiological boundary between transient negative affect and pathological depression. As highlighted in recent technical studies [9,10], many approaches rely exclusively on single-domain features (e.g., spectral power), thereby ignoring the intrinsic coupling between spatial asymmetry and temporal dynamics. In depression recognition, spatial asymmetry (such as frontal *α*-imbalance) reflects the stable trait-like disruption of emotional regulation, while temporal dynamics (such as non-linear complexity) capture the adaptability of neural information processing. The neglect of these inter-domain correlations restricts the model’s ability to capture the heterogeneous neurophysiological fingerprints of the depressive brain, leading to limited recognition accuracy.

However, relying on a single feature domain fails to capture the heterogeneous neurophysiological alterations associated with depression. Specifically, in the frequency domain, depression is typically marked by elevated *α*-wave power and suppressed *β* activity; however, capturing these variations remains challenging due to the non-stationary nature of EEG signals when using traditional Fourier transforms. From a spatial perspective, the ‘Frontal Asymmetry Hypothesis’ highlights a distinctive reduction in left-frontal activity (linked to approach motivation) coupled with relative right-frontal hyperactivity. Furthermore, non-linear analysis reveals that pathological systems often suffer from a ‘Loss of Complexity’, which is manifested as lower EEG entropy values compared to healthy controls. Consequently, fusing frequency, spatial, and non-linear features is indispensable for constructing a holistic representation of the depressive brain.

To address these challenges, this study presents a unified framework, the Multi-Head Additive Attention Network, designed to distinguish between transient negative affect and pathological depression. The primary innovation lies in the construction of a heterogeneous feature space that simultaneously characterizes frequency oscillations, spatial asymmetry, and non-linear complexity. Diverging from traditional concatenation strategies that treat all dimensions equally, our approach introduces a multi-head attention mechanism to dynamically recalibrate the importance of each feature channel. This enables the model to autonomously prioritize depression-specific neurophysiological signatures. Furthermore, we constructed a composite cross-phenotypic dataset by integrating negative emotion samples from DEAP and depression data from HUSM. The proposed framework achieves a recognition accuracy of 92.65%, offering a solution for early clinical screening.

## 2. Materials and Methods

### 2.1. Emotional Modelling Strategy

**Figure 1:**
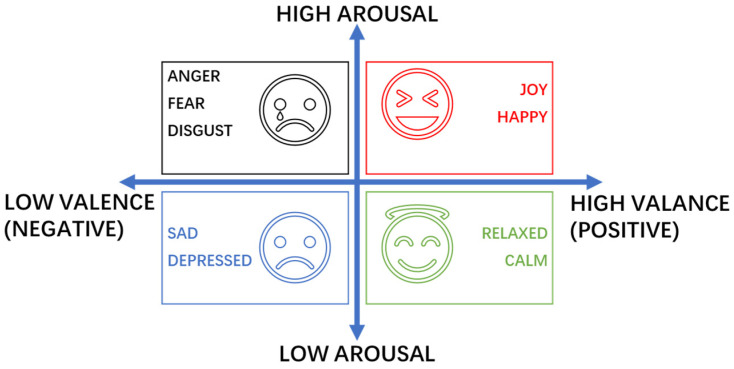
Diagram of the Two-dimensional VA Emotional Model.

There are two main types of existing quantitative models of emotions: discrete and continuous models. Traditionally, emotions are viewed as independent categories, such as happiness, sadness, and anger, which are isolated from one another. However, advancing research increasingly recognizes the complexity and continuity of human affect, suggesting that discrete models provide a limited representation. To address this, the two-dimensional Valence-Arousal (VA) model was adopted. This model defines emotions along two dimensions: valence and arousal. The horizontal axis represents the valence dimension, denoting the polarity of the emotional state, ranging from negative (e.g., sad, upset) to positive (e.g., happy, joyful). The vertical axis represents the arousal dimension, reflecting the intensity of the emotion from weak to strong. Through this framework, various complex emotions can be expressed as linear combinations of these two dimensions, thereby effectively capturing the continuous dynamics of emotional change. Figure 1 shows the schematic diagram of the VA emotional model.

In this study, due to the need for detailed classification of negative emotions, it is necessary to better reflect the process of emotional change, and the emotion classification of the discrete emotion model cannot be divided into the specific emotional state of a person, so this paper chooses the continuous emotion model.

### 2.2. Data Acquisition and Preprocessing

#### 2.2.1. DEAP Dataset

The DEAP dataset was experimentally acquired by Koelstra et al. in collaboration with Queen Mary University of London (UK), the University of Twente (Netherlands), the University of Geneva (Switzerland), and the Swiss Federal Institute of Technology to study multichannel data on human emotional states. The EEG signals were recorded using 32 channels positioned according to the International 10–20 electrode system at a sampling frequency of 512 Hz [11]. The dataset involves 32 participants (16 males and 16 females, aged 19–37, mean age = 26.9). All participants were right-handed, with normal or corrected-to-normal vision and normal hearing, and reported no history of mental or neurological disorders. Thirty-two participants viewed 40 music video clips of their choice. Subsequently, they assessed their levels of arousal, valence, dominance, and liking using the Self-Assessment Manikin (SAM). Participants assigned scores ranging from 1 to 9 for each clip, with 1 representing sadness/calmness and 9 representing happiness/excitement. In this paper, we utilize the preprocessed version of the dataset. In this version, the sampling rate was downsampled to 128 Hz, and a bandpass filter of 4–45 Hz was applied to remove high-frequency noise, retaining the informative EEG frequency bands. To mitigate artifacts caused by physiological noise (such as ocular, EMG, and ECG signals), the preprocessing pipeline involved identifying and removing signal outliers, followed by common average referencing. In this paper, we use the preprocessed DEAP dataset to classify EEG emotions [12].

**Figure 2:**
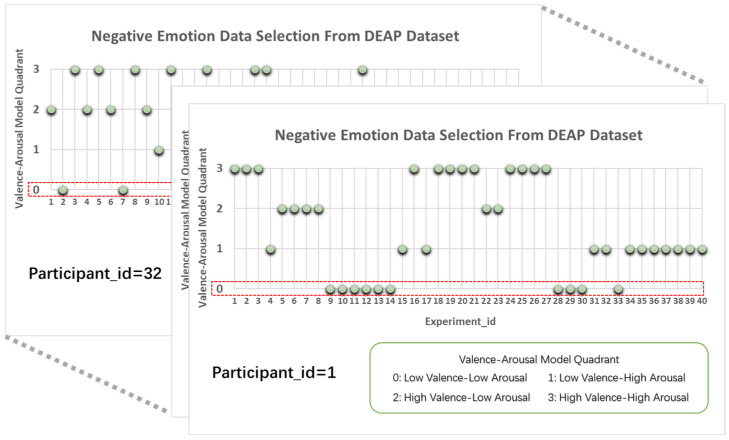
Negative Emotion Data Selection from DEAP Dataset.

The DEAP dataset was analyzed based on the Valence-Arousal (VA) emotional model. While traditional discrete emotion hierarchies categorize emotions into six broad classes (joy, sadness, anger, fear, surprise, and disgust), basic emotions associated with negative emotions—specifically sadness, fear, and disgust—are intrinsically linked to depression. On the VA model, these emotions are predominantly concentrated in the region of low pleasantness, specifically the “left half” where Valence < 5. Lower valence values correspond to more severe grades of negative emotion, which are more prone to inducing depressive states. Furthermore, arousal levels correspond to distinct depressive manifestations: low arousal typically manifests as “ordinary depression” (e.g., lethargy), whereas high arousal manifests as “anxious or irritable depression”, characterized by anxiety and tension. Consequently, based on the VA model within the DEAP dataset, this study utilizes the valence dimension to stratify negative moods and investigate their intrinsic relationship with depression, as shown in Figure 2.

#### 2.2.2. HUSM Depression Dataset

The depression EEG data used in this paper was provided by Hospital Universiti Sains Malaysia (HUSM). Thirty-four outpatients (17 males and 17 females, mean age = 40.3 ± 12.9 years) diagnosed with Major Depressive Disorder (MDD) were recruited in accordance with the experimental design approved by the Human Ethics Committee of HUSM. All subjects were right-handed and reported no history of other neurological disorders. All subjects provided written informed consent after fully understanding the experimental procedures. The enrolled patients with MDD met the internationally recognized diagnostic criteria for depression, namely the DSM-IV [13].

The EEG acquisition equipment followed the International 10–20 system. Data were recorded using a linked ear (LE) reference and digitized at a sampling rate of 128 Hz. The pre-filter range was set to 4–45 Hz, with an additional 50 Hz notch filter applied to suppress power line noise [14]. Eyes-closed resting-state data were utilized in this study. In this paradigm, subjects were instructed to sit in a semi-recumbent position with minimal head movement, and data were recorded for five minutes with their eyes closed.

#### 2.2.3. Channel Selection

EEG signals comprise distinct frequency components, conventionally categorized into *δ* (0.5–3 Hz), *θ* (4–7 Hz), *α* (8–13 Hz), *β* (14–30 Hz), and *γ* (>30 Hz) bands. In the context of affective computing and depression recognition, *α* and *β* oscillations serve as the most critical spectral biomarkers [15]. Neurophysiologically, *α*-activity reflects cortical inhibition, while *β*-activity is associated with alertness, cognitive load, and anxiety. Although *α*-rhythms are predominantly generated in the occipital region during resting states, Frontal Alpha Asymmetry (FAA)—the power difference between left and right frontal hemispheres—is a well-established indicator of emotional regulation and depressive pathology. Depression is typically characterized by hypoactivation in the left frontal lobe (increased *α* power) and hyperarousal in the right frontal lobe (increased *β* activity) [16].

**Figure 3:**
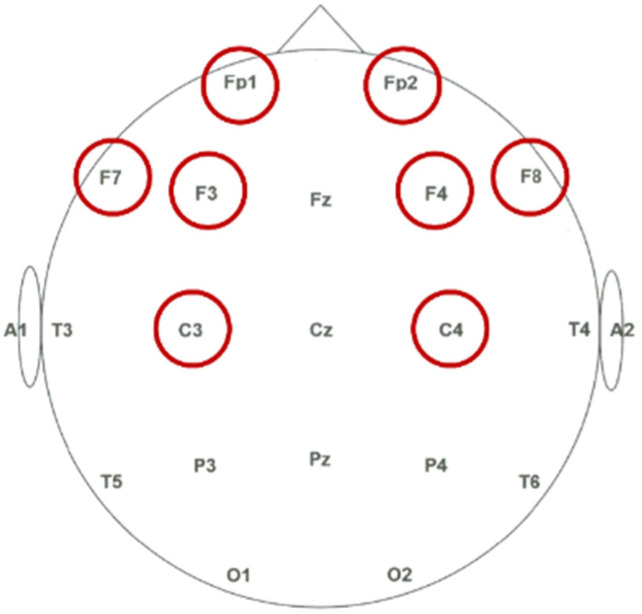
Selected Channels Marked by Red Circles from the EEG 10–20 Electrode System.

Consequently, to capture these emotion-regulation circuits, the prefrontal and central regions were prioritized over the visual occipital cortex. Specifically, eight discriminative channels covering the Prefrontal (Fp1, Fp2), Frontal (F3, F4, F7, F8), and Central (C3, C4) areas were selected to extract the spatial asymmetry and spectral power features. The spatial distribution of these electrodes is illustrated in Figure 3.

#### 2.2.4. Cross-Phenotypic Fusion EEG Dataset

To delineate the intrinsic physiological boundaries between transient negative affect and clinical depression, we constructed a unified repository termed the “Cross-Phenotypic Fusion EEG Dataset.” This nomenclature highlights the integration of two distinct affective phenotypes: non-pathological negative emotional states (physiological response) and pathological depressive traits (clinical disorder). To ensure affective validity and class balance, rigorous inclusion criteria were applied. For the negative emotion subset (Control Group), we restricted selection to DEAP trials with low valence (<2.5) and low arousal (<5), analyzing only steady-state data (15–60 s) to mitigate transient instability. This yielded 510 valid negative emotion samples. Conversely, for the depression subset (Experimental Group), although the HUSM dataset provided a larger volume of resting-state data, utilizing the full set would introduce severe class imbalance. To address this, we performed random down-sampling on the HUSM pool, selecting 510 depression samples to strictly maintain a 1:1 balanced ratio.

**Figure 4:**
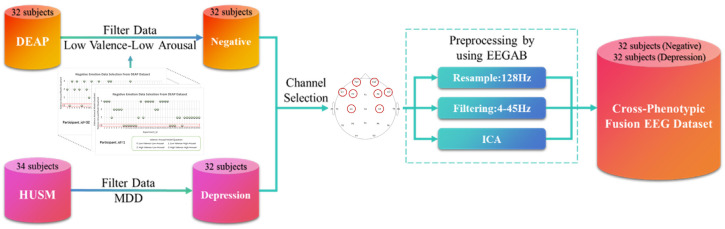
Cross-Phenotypic EEG Data Fusion and Preprocessing Process.

To ensure signal consistency across these heterogeneous sources, a unified preprocessing pipeline was applied to the HUSM data using the EEGLAB toolbox in MATLAB R2021b. First, a bandpass filter of 4–45 Hz was applied to remove high-frequency noise and power line interference. Subsequently, Independent Component Analysis (ICA) was performed based on the InfoMax principle [17] to identify and remove ocular and muscle artifacts [18]. Finally, the sampling frequency was downsampled to 128 Hz to match the preprocessed DEAP dataset. Consequently, data from both sources were aligned to a unified tensor format of 8 channels × 960 time points (7.5 s × 128 Hz), resulting in a final dataset of 1020 samples for the subsequent multi-domain feature extraction and attention-based classification modules, as shown in Figure 4. 

### 2.3. Overall Framework

**Figure 5:**
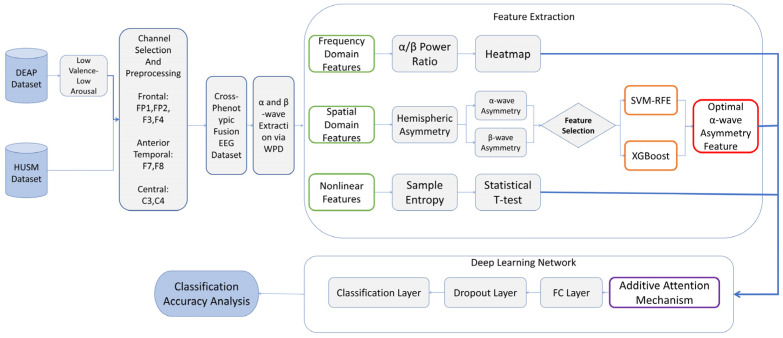
Schematic diagram of the proposed Multi-Head Additive Attention Network framework. The pipeline comprises three key stages: (1) Multi-domain feature extraction, where frequency (WPD), spatial (Asymmetry), and non-linear (Sample Entropy) features are extracted in parallel; (2) Adaptive feature fusion, utilizing a Multi-Head Additive Attention mechanism to dynamically reweight feature importance; and (3) Classification, which outputs the probability of depression versus negative emotion.

To effectively distinguish between pathological depression and transient negative emotions, we propose a comprehensive depression recognition framework, termed Multi-Head Additive Attention Network (MHA-Net). As illustrated in Figure 5, the framework operates through a hierarchical pipeline consisting of three key stages:

### 2.4. Multi-Domain Feature Extraction

#### 2.4.1. Frequency Domain Features Based on WPD

**Figure 6:**
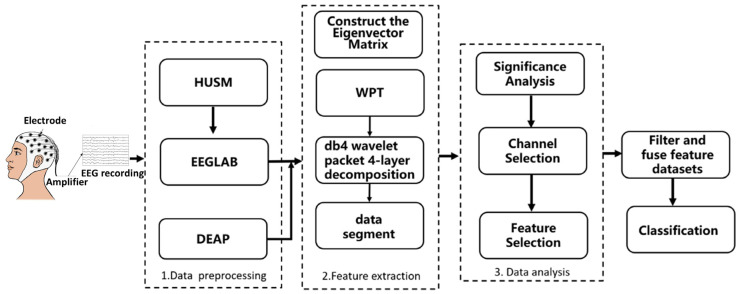
Schematic diagram of the frequency domain feature extraction pipeline based on WPD.

Building upon the standardized Cross-Phenotypic Fusion Dataset, this study implements a comprehensive feature extraction pipeline. First, a four-layer Wavelet Packet Decomposition (WPD) is applied to the preprocessed signals to isolate emotion-sensitive frequency bands [19]. Based on this decomposition, three distinct categories of discriminative features—frequency (*α*/*β* PSD ratio), spatial (asymmetry), and non-linear (sample entropy)—are extracted to construct the final multi-domain feature matrix. Subsequently, significance analysis is conducted to identify the channels with the highest discriminative power. These selected features are then fused to serve as the input for the subsequent attention-based classification network. The overall workflow is illustrated in Figure 6.

#### 2.4.2. PSD Ratio of *α*-Wave to *β*-Wave

The basic dimensions of EEG signal analysis are time-domain and frequency-domain features. PSD, as an intuitive method for frequency-domain analysis, can directly reflect the activity of EEG signals in different frequency bands and is often used as a significant feature in EEG research. According to previous studies, the *α*-wave is positively correlated with positive emotional activation, while the *β*-wave’s power changes are coupled with the generation of negative emotions; *β*-wave power, in particular, increases significantly during negative emotional states. Therefore, this study adopts the *α*-wave/*β*-wave PSD ratio as one of the core indicators for feature extraction, calculating the PSD of the *α*-wave and *β*-wave bands respectively and using their ratio to quantify the emotional state.

Compared to approaches relying on static single-band power, the *α*/*β* PSD ratio provides a more robust characterization of the neural dynamics underlying negative emotions. Single-band features are inherently susceptible to non-pathological artifacts, such as inter-subject variability in baseline power and signal fluctuations caused by acquisition devices. As a relative metric, the *α*/*β* ratio effectively normalizes these absolute variations, thereby enhancing feature consistency and cross-subject generalization. Furthermore, this ratio tracks the continuous trajectory of emotional fluctuation rather than merely identifying static states. Crucially, it serves as a quantitative index of emotional intensity: a lower ratio—resulting from suppressed *α*-inhibition and elevated *β*-arousal—indicates a more severe negative emotional state.

**Figure 7:**
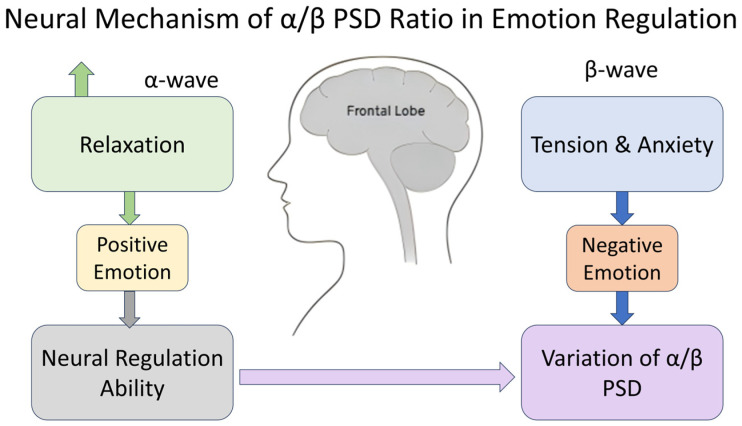
Neural mechanisms of emotion regulation.

From a neurophysiological perspective, the *α*-wave is closely related to inhibitory neural activity in the cerebral cortex and typically dominates during resting, relaxed, or emotionally stable states, reflecting the brain’s emotional control capabilities. The *β*-wave, originating mainly from the frontal and central regions, is primarily responsible for cognitive load and emotional arousal; its power increase is often associated with anxiety, tension, or the generation of negative emotions. In individuals in negative emotional states or those with depression, the over-activation of *β*-waves may lead to dysfunction in the prefrontal cortex’s emotional regulation, causing attentional resources to focus excessively on negative information. This is accompanied by a reduction in *α*-wave inhibitory function, resulting in a decrease in the *α*-wave/*β*-wave power ratio. This decrease in the *α*-wave/*β*-wave ratio is also considered to be related to abnormal neural activity in emotion-related brain regions such as the limbic system and anterior cingulate cortex, manifesting as hypersensitivity to negative stimuli and weakened regulatory ability. Therefore, the *α*-wave/*β*-wave PSD ratio is not only a sensitive biomarker for emotional states but also reflects the imbalance in the brain’s emotional regulation and executive functions in depression from a neural mechanism level, as shown in Figure 7. This indicator has been validated in multiple studies on depression, anxiety, and emotional disorders. Research indicates that enhancing *α*-wave activity while reducing *β*-wave activity is beneficial for alleviating negative emotions and fatigue, restoring attention, and plays a positive role in maintaining emotional stability. Combined with the neuroscientific explanations and EEG research mentioned above, the rationale for the *α*-wave/*β*-wave PSD feature is confirmed.

**Figure 8:**
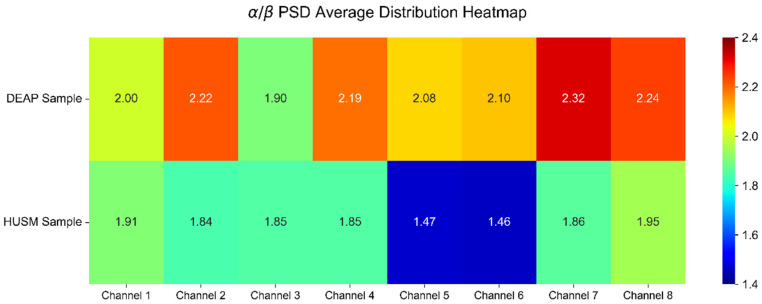
*α*/*β* PSD heat map.

Figure 8 shows the average distribution of the *α*/*β* PSD ratio for the DEAP and HUSM sample groups across eight selected EEG channels. The horizontal axis represents the selected channels, numbered 1 through 8 (corresponding to Fp1, Fp2, F3, F4, F7, F8, C3, and C4), while the vertical axis represents the two sample sources. The numerical value within each cell indicates the average value for that channel within the corresponding sample group. The heatmap coloration reflects magnitude: colors trending toward red signify a higher ratio, corresponding to a more intense emotional state, whereas colors trending toward blue indicate a lower ratio. It is clearly observable from the figure that the *α*/*β* PSD ratios of the DEAP samples are significantly higher than those of the HUSM samples across all eight channels. This disparity is most pronounced in the channels located near the frontal lobe (F7 and F8) and the central region (C3 and C4). This difference indicates that the activation level of negative emotion in the HUSM samples is markedly reduced in these areas, reflecting a systemic difference in the frequency-domain EEG features between the two sample groups.

To further demonstrate the feature’s effectiveness, this paper employs a non-parametric test—the Mann–Whitney U test—to compare whether a significant difference exists between the samples in the DEAP and HUSM datasets. The Mann–Whitney U test is a non-parametric method that does not require the data to adhere to a normal distribution, making it suitable for small samples or non-normally distributed data, which aligns with the characteristics of the current data. Furthermore, this method possesses strong robustness and flexibility. Its significance result is represented by *p*-value; when the *p*-value is less than the significance level of 0.05, it can be concluded that a significant difference exists between the two data groups.

As can be seen from Table 1, among the 8 channels adopted in this paper, only the F4 channel had a significance level slightly higher than 0.05, while the test results for the *α*/*β* PSD feature in all other channels met the standard. This result indicates that the selected feature possesses good discriminatory power for classification, and the significant difference confirms that it can serve as one of the multi-domain features.

#### 2.4.3. Left-Right Brain Asymmetry

Hemispheric asymmetry in frontal EEG is closely linked to distinct emotional traits. According to the approach-withdrawal hypothesis, left and right frontal activations govern positive and negative affect, respectively. This asymmetry serves as a robust indicator for both transient mood changes and stable pathological states like depression. By adopting a normalized *α*-PSD asymmetry approach [20], we effectively neutralized individual power differences to accurately quantify the differential activation between hemispheres

#### 2.4.4. Sample Entropy

EEG signals are inherently non-stationary and chaotic, characterized by significant non-linear dynamics. Traditional linear analysis methods often fail to capture this complexity, potentially resulting in the loss of critical physiological information. Conversely, non-linear features have been shown to provide a more realistic and objective representation of emotional state transitions [21]. Sample Entropy, a robust measure of time-series complexity, was introduced to quantify these non-linear characteristics. Compared to other entropic measures, SampEn is independent of data length and exhibits superior resistance to noise and interference, making it particularly suitable for analyzing complex EEG signals [22]. A higher SampEn value indicates greater signal complexity and elevated cortical activation.

It can be seen that the algorithm of sample entropy mainly contains three parameters, which are the length of the data N, the embedding dimension m, and the effective threshold r. The following describes the selection process of the sample entropy parameters:

(a) m is the embedding dimension, which is the length of the window set when calculating the sample entropy, in general, the value of m is usually set to 1 or 2. When the value of m is greater than 2, the required data length N needs to be very long, and only when N is greater than 5000 will it be very good, but in this case, the required r will also be very large, and the analyses of the signal sequences will result in a large amount of information loss. Therefore, in this paper, the value of m is chosen to be 2.
(b) r is the effective threshold, which indicates the similarity tolerance, when the value of r is large, more information will be lost, and when the value of r is small, the statistical properties of the system will not be estimated ideally. The conclusion is that usually when the value of r is taken between 0.1 and 0.25 SD(x) (SD(x) is the standard deviation of the sequence), the feature parameter representation is more desirable. In this paper, we choose 0.2* SD(x) as the value of r.
(c) The parameter N denotes the length of the data, and there is no restriction on the selection of N. The study points out that the number of data points should be controlled in the region of 100 to 5000 to derive the desired statistical features.

**Figure 9:**
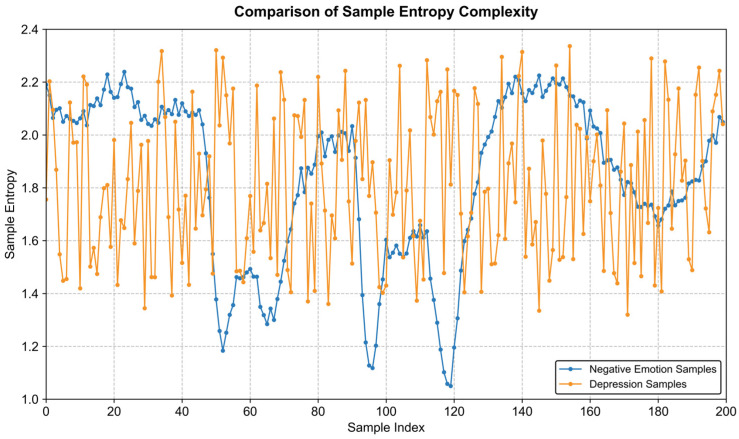
Comparison of sample entropy complexity.

**Figure 10:**
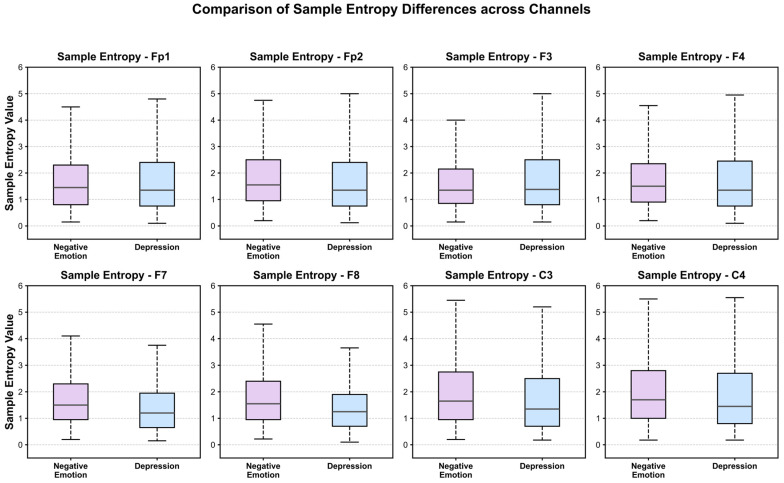
Significant differences in sample entropy.

Sample Entropy was introduced as a key non-linear metric to quantify the complexity of EEG signals. This approach is grounded in neuroscientific findings suggesting that depression is characterized by a reduction in neural complexity, manifesting as more stable and monotonous brain activity. Our analysis, visualized in Figure 9 and Figure 10, corroborates this hypothesis. Specifically, samples associated with negative emotions (higher volatility) exhibit consistently higher entropy values compared to the depression samples. To statistically validate the discriminative power of this feature, an Independent-samples T-test was conducted with a significance level of *α* = 0.05. The results confirmed that the sample entropy feature achieved statistical significance across all eight selected channels (*p* < 0.05).

The distributional characteristics are further elucidated by the boxplots in Figure 10, where a distinct separation between the two groups is observable across all channels. The depression group consistently exhibits a trend of diminished sample entropy, characterized by lower median values and more concentrated distributions (i.e., narrower interquartile ranges). This reduction indicates a decline in the non-linear complexity of brain activity during depressive states, suggesting a shift towards neural synchronization and rigidity, which aligns with existing literature on depressive EEG patterns. Conversely, the negative emotion group displays relatively higher sample entropy, reflecting greater signal volatility and preserving a degree of dynamic regulatory capability.

Collectively, these results demonstrate that the sample entropy feature effectively captures alterations in cerebral neural dynamics and possesses robust discriminatory capability. In summary, sample entropy is not only statistically significant but also serves as a critical non-linear complement to the frequency-domain and spatial-domain features, thereby enhancing the comprehensiveness of the subsequent classification framework.

**Figure 11:**
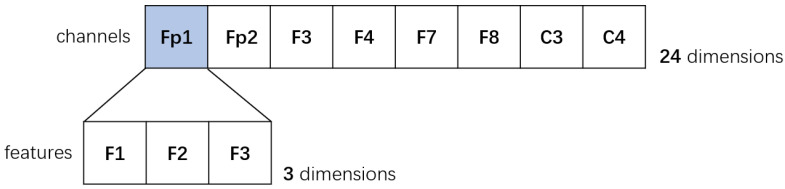
Distribution of 24-dimensional feature matrix.

After extracting the frequency-domain (F1), spatial-domain (F2), and non-linear (F3) features, the final feature matrix was constructed. As detailed in Section 2, the screening process yielded 510 samples that met the criteria for severe negative emotion (from DEAP), and 510 samples were selected from the depression group (from HUSM) to ensure a balanced dataset. For each of these 1020 total samples, the 3 aforementioned features (F1: *α*/*β* PSD ratio; F2: *α*-wave left-right brain asymmetry; F3: sample entropy) were calculated for all 8 selected channels. This process generated a final matrix of 1020 × 24 (1020 samples × 24 features), which serves as the definitive input for the subsequent classification. The structure of each feature vector is shown in Figure 11.

### 2.5. Feature Fusion and Recognition Based on Attention Mechanism

#### 2.5.1. Multi-Head Additive Attention Mechanism

This section constructs a classification model that integrates the multi-domain features, introducing a Multi-Head Additive Attention mechanism to perform adaptive weighted learning on the feature dimensions. It further conducts feature fusion and, based on this fusion model, proceeds with classification training, test set validation, and multi-dimensional performance analysis.

To synergize the distinct feature modalities, we implemented a Multi-Head Additive Attention mechanism, which is particularly robust for processing low-dimensional, non-sequential data. Structurally, the 24-dimensional input vector is partitioned into three 8-dimensional subspaces, corresponding to the frequency (F1), spatial (F2), and non-linear (F3) domains, implementing domain-specific feature weighting. These subspaces are processed in parallel by three independent attention heads to adaptively capture domain-specific feature importance. Each head learns a unique weight vector (*α*) using a fully connected layer and a Softmax activation (Formulas (1) and (2)).

(1) $$\alpha \;=\;softmax(W_{\alpha }\cdot x_{\mathrm{concat}}+b_{\alpha })$$

(2) $$\mathrm{Softmax}\left(z_{i}\right)=\frac{e^{z_{i}}}{\sum_{j=1}^{n}e^{z_{j}}}\;\mathrm{for}\;i=1,2,\ldots ,n$$

**Figure 12:**
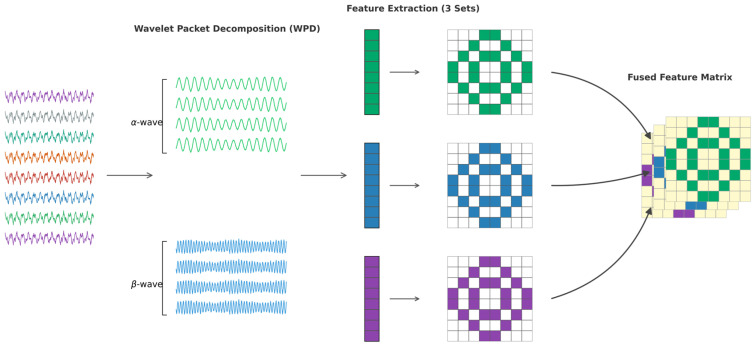
Schematic illustration of the Multi-Domain Feature Fusion framework. The framework synergizes heterogeneous EEG representations, including frequency (PSD ratio), spatial (normalized -asymmetry), and non-linear (Sample Entropy) features. These concatenated high-dimensional features are projected into a latent subspace, the Multi-Head Additive Attention mechanism achieves domain-specific feature weighting, thereby adaptively recalibrating the contribution of each domain for depression recognition.

Finally, the normalized weight vector and the input feature matrix are multiplied element-by-element to obtain the weighted fusion features, and three new sets of weighted feature vectors are formed. Finally, the three groups of weighted results are spliced into a 72-dimensional fusion matrix, the resulting 72-dimensional concatenated vector is fed into a fully connected layer (16 neurons, ReLU activation) followed by a 0.4 dropout layer. The network is trained using the Adam optimizer (learning rate: 0.001, batch size: 30), underscoring the lightweight nature of the framework, with approximately 1.8 k trainable parameters and an average inference time of <1 ms per sample (tested on a standard CPU), the proposed framework demonstrates high computational efficiency, underscoring its lightweight nature. As shown in Figure 12, which is convenient for further modeling and classification of the subsequent self-attention module. The final model formula can be expressed as follows.

(3) $$x_{fused}=\alpha ⊙x_{\mathrm{concat}}$$

#### 2.5.2. Ablation Study of Attention

**Figure 13:**
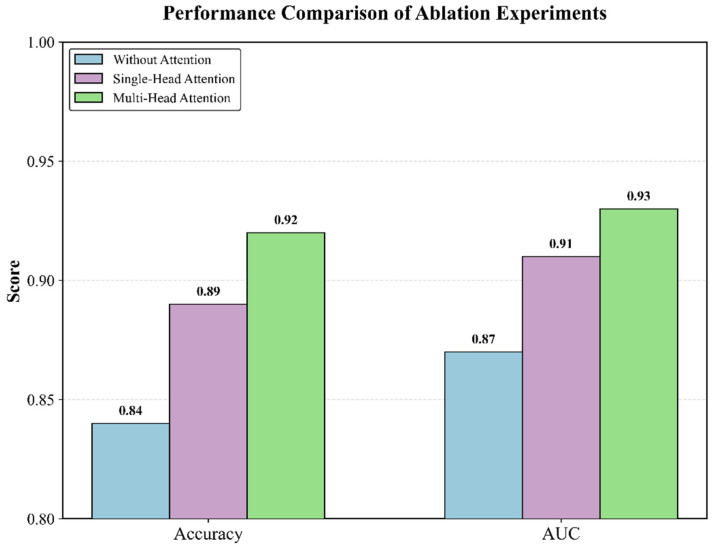
Comparison of attentional mechanism ablation experiments.

To validate the effectiveness of this multi-head design, an ablation study was performed (Figure 13). We compared three models under identical training parameters: (1) No Attention (features directly concatenated), (2) Single-Head Attention, and (3) our proposed Multi-Head Additive Attention.

As shown in Figure 13, the Multi-Head model achieved the best results in both accuracy (0.92) and AUC (0.93), significantly outperforming the Single-Head (Acc: 0.89, AUC: 0.91) and No-Attention (Acc: 0.84, AUC: 0.87) models. This confirms that the multi-head structure is superior for capturing diverse patterns across the different feature sub-spaces.

These results indicate that while the introduction of an attention mechanism improves overall performance, the multi-head approach is more effective. By introducing multiple independent weight parameters to model features in different subspaces, the multi-head mechanism can better enhance the model’s overall classification performance, thus validating the effectiveness of the proposed method for feature weighting and fusion.

#### 2.5.3. Experimental Implementation

All experiments were implemented using the PyTorch (version 2.3.0) framework on a workstation equipped with an Intel Core Ultra 7 265K processor and an NVIDIA GeForce RTX 5060 Ti GPU. For the proposed model, a 5-fold cross-validation scheme was strictly applied to ensure robust performance evaluation. In each fold, the dataset was partitioned into 80% for training and 20% for testing. The network was optimized using the Adam optimizer with a binary cross-entropy loss function. To mitigate overfitting, a Dropout rate of 0.4 was applied, and the training was conducted for 30 epochs, with convergence typically observed around the 22nd epoch.

To benchmark the proposed method, Support Vector Machine (SVM) [23,24] and eXtreme Gradient Boosting (XGBoost) [25] were employed as baseline classifiers. Additionally, a Recurrent Neural Network (RNN) [26] was utilized to evaluate the impact of sequential modeling. Unlike standard validation, we implemented a Nested Cross-Validation (NCV) strategy for the SVM to eliminate bias in hyperparameter selection. This involved an outer 5-fold loop to estimate generalization error and an inner 3-fold loop to perform a grid search for the optimal penalty parameter (C) and RBF kernel coefficient (*γ*). For XGBoost, the model was configured to optimize the logistic loss for binary classification.

Furthermore, to statistically validate the superiority of the selected features and models, the DeLong test was utilized to compare the differences between Receiver Operating Characteristic (ROC) curves. A *p*-value of less than 0.05 was considered statistically significant.

## 3. Results and Discussion

### 3.1. Evaluation of Spatial Features and Baseline Classifiers

**Figure 14:**
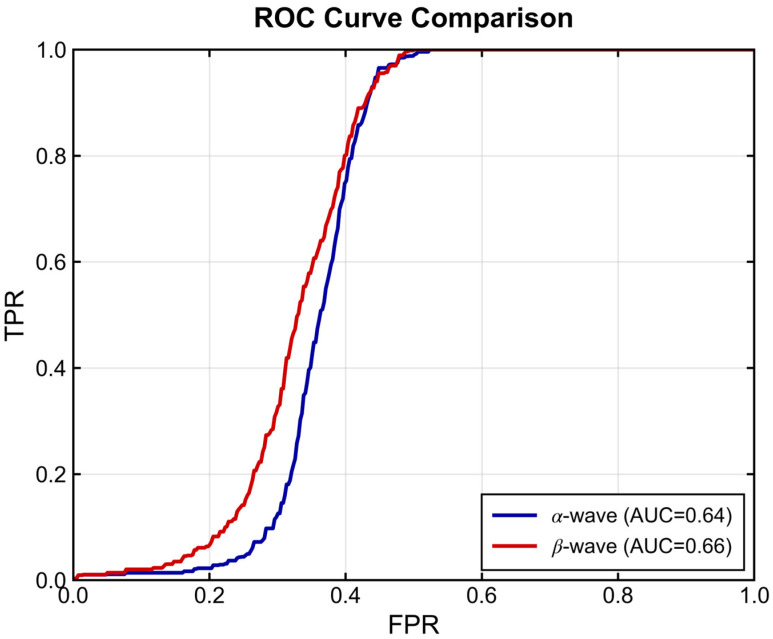
ROC curves obtained from SVM classifier using nested cross-validation.

**Figure 15:**
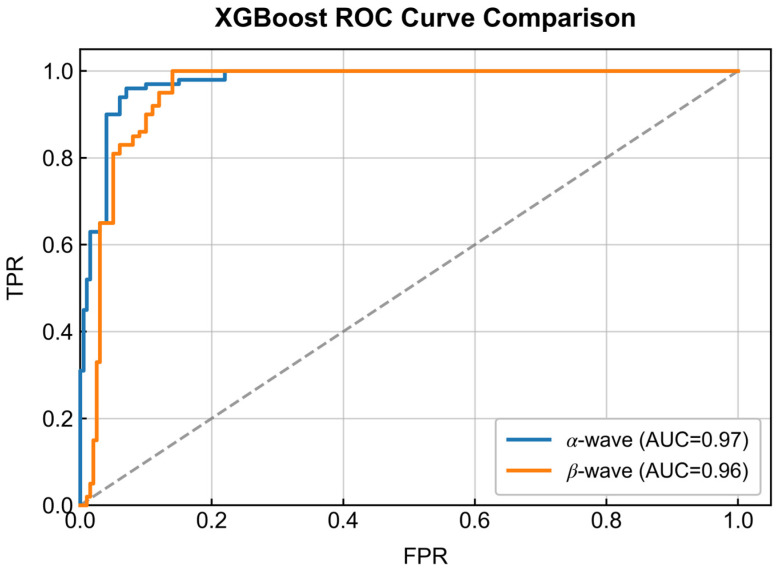
ROC curves obtained from the XGBoost classifier. The *α* -wave feature (blue line) demonstrates superior discriminative performance.

To determine the optimal frequency band for depression recognition and establish a performance baseline, we evaluated the discriminative power of the extracted spatial features—specifically, the inter-hemispheric asymmetry of *α*-waves versus *β*-waves—using SVM and XGBoost classifiers. Figure 14 and Figure 15 visualize the Receiver Operating Characteristic (ROC) curves derived from the test set. As illustrated in Figure 14, the SVM classifier yielded suboptimal performance, with curves hovering closer to the diagonal line compared to the non-linear model. This suggests that the linear decision boundaries of SVM are insufficient to capture the complex physiological dynamics inherent in depressive EEG signals.

In contrast to the linear limitations of SVM, the XGBoost framework (Figure 15) demonstrated a substantial performance leap by modeling non-linear feature interactions. To quantify this improvement, Table 2 details the precise performance metrics. As indicated in the table, XGBoost improved classification accuracy by approximately 18 percentage points over SVM across both frequency bands. While SVM accuracies ranged from 70.49% to 73.14%, XGBoost achieved accuracies exceeding 89%, confirming the necessity of non-linear modeling for this task.

Further analysis of Table 2 reveals a critical distinction between the frequency bands. The *α*-wave asymmetry feature achieved the highest overall performance, with an accuracy of 91.14% and an AUC of 0.97. Although the numerical superiority over the *β*-wave appears marginal (AUC: 0.97 vs. 0.96), the *α*-wave ROC curve exhibits a steeper ascent in the low False Positive Rate (FPR) region, implying higher sensitivity which is vital for clinical screening. To rigorously verify this disparity, a DeLong test was conducted. The results confirmed that the discriminative superiority of the *α*-wave feature is statistically significant (Z = 3.45, *p* < 0.001). Consequently, *α*-wave asymmetry was identified as the most robust spatial feature and was selected for the subsequent construction of the attention-based neural network.

### 3.2. Analysis of Depressive Mood Recognition Results

**Figure 16:**
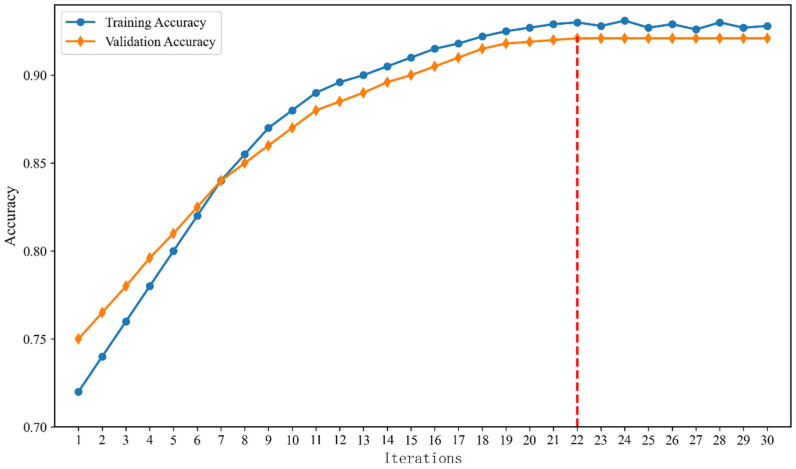
The model learns the global fitness change.

After the aforementioned training of the attention-based neural network, the model’s classification performance was analyzed. As illustrated in Figure 16, the model exhibited a sharp increase in accuracy during the initial 10 iterations, with the training accuracy surging from approximately 72% to 88%. This rapid ascent indicates the model’s efficiency in capturing salient feature patterns in the early stages. As the iterations progressed, the convergence rate gradually stabilized. Notably, the validation accuracy closely tracked the training curve, reaching a stable peak of 92.2% with minimal divergence from the training accuracy. This tight alignment suggests that the model possesses robust generalization capabilities and effectively avoids overfitting. Furthermore, the final model accuracy improved by 22% compared to the initial accuracy. This demonstrates that the proposed multi-head additive attention learning model can effectively enhance the accuracy of discriminating between depression and negative emotions, and that the model continuously iterates to search for higher accuracy. Additionally, compared to traditional deep learning methods, the multi-head additive attention learning model proposed in this study not only maintains high accuracy but also possesses better real-time performance. The model has low computational complexity during the inference phase and requires fewer iterations, enabling it to complete the recognition of emotional states and depression states in a short time, making it suitable for classification scenarios in practical medical applications.

**Figure 17:**
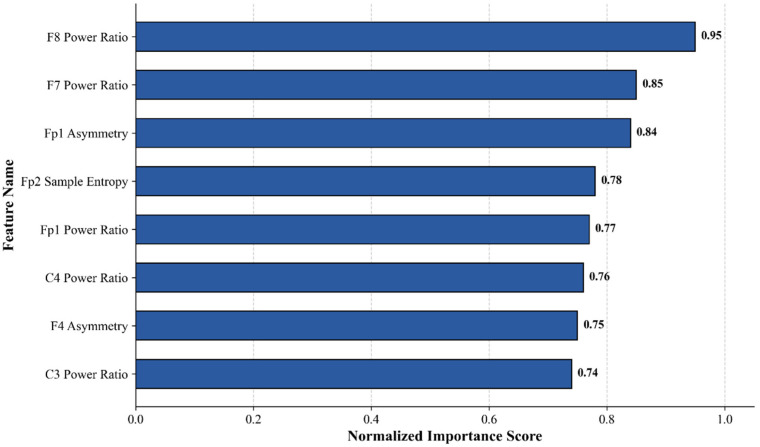
Feature importance ranking.

Figure 17 displays the top 8 most important features selected from the 24-dimensional raw features, along with their normalized importance scores. Figure 17 highlights the top 8 most discriminative features ranked by their normalized importance scores. The F8 power ratio emerged as the most critical predictor, achieving a normalized score of 0.95. This finding underscores the pivotal role of right prefrontal cortical activity in differentiating depressive states. Neurophysiologically, this aligns with established pathology regarding frontal alpha asymmetry and prefrontal hypoactivation in depression. The prominence of the F8 feature suggests that our model successfully captures the intrinsic neuropathological signatures of depression, enhancing its clinical interpretability. As can also be seen from Figure 8, the relative difference in the F8 channel’s power ratio between the two datasets was the largest, reaching 30.5%, exceeding all other channels. Subsequently, features such as the F7 power ratio, Fp1 asymmetry, Fp2 sample entropy, Fp1 power ratio, C4 power ratio, F4 asymmetry, and C3 power ratio also exhibited high normalized scores. Among the top 5 features, frequency-domain power features are predominant, suggesting that changes in energy distribution across different EEG frequency bands are the core source of information for identifying the subject’s state, further illustrating the key role of EEG frequency-domain features in state recognition, such as emotion. However, this also demonstrates the supplementary value of inter-brain activity differences and non-linear features in the classification process, which can provide complementary information from different dimensions to aid discrimination.

It is particularly noteworthy that these high levels of statistical significance were maintained despite the inherent challenges of the cross-phenotypic design, specifically the demographic differences (age gap) and varying acquisition paradigms (stimulus-induced vs. resting-state) between the cohorts. Instead of being mere artifacts of these confounding variables, the resilience of these key features under such a rigorous “stress test” strongly suggests that they capture the intrinsic, resilient pathological signatures of clinical depression that transcend situational noise. This robustness serves as a reliable physiological foundation for accurate classification.

After the overall model construction and parameter iteration were complete, the performance was then evaluated on the entire sample dataset. To more comprehensively reflect the model’s performance, confusion matrices were plotted for the test set and the entire dataset. A confusion matrix can intuitively reflect the classification results and can also be used to derive multi-dimensional metrics such as precision, recall, and F1-score, allowing for a more thorough analysis of the model’s performance.

**Figure 18:**
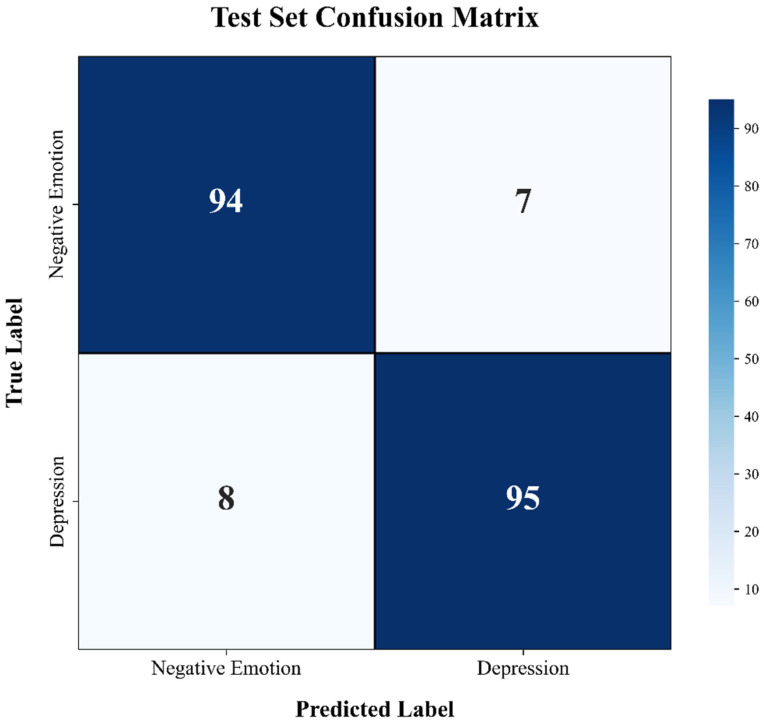
Confusion matrix illustrating the classification performance on the independent test set.

**Figure 19:**
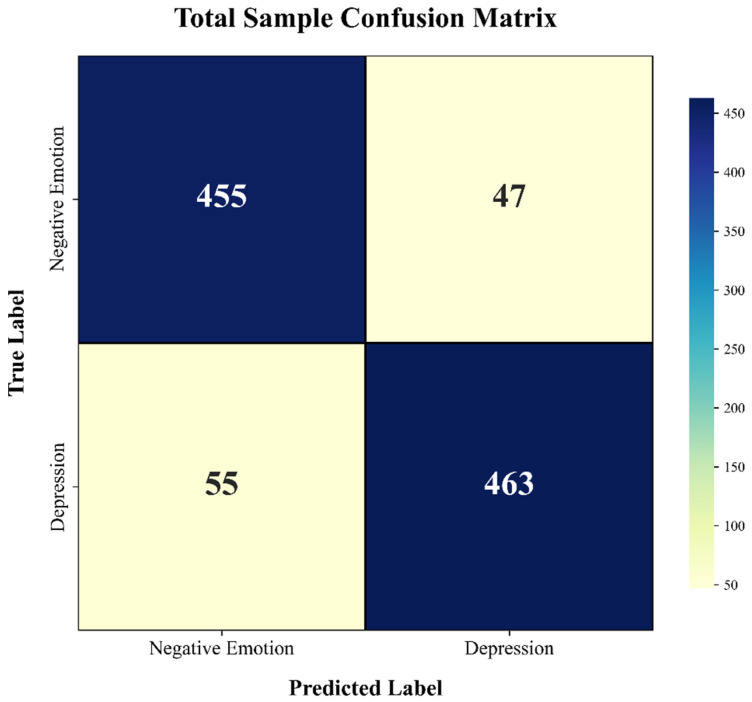
Confusion matrix illustrating the classification performance on the total dataset.

Figure 18 and Figure 19 present the confusion matrices for the independent test set and the total dataset, respectively. As quantified in Figure 18, the model demonstrated robust classification performance on the test set, correctly identifying 93% of the Negative Emotion trials (94/101) and 92% of the Depression trials (95/103). To verify the generalization capability of the model, this evaluation was extended to the entire dataset (Figure 19). The classification patterns in the total sample closely mirrored those of the test set, confirming the stability of the proposed framework. Crucially, the off-diagonal misclassification rates in both matrices exhibit a symmetric distribution (e.g., 7 vs. 8 in the test set), indicating that the model possesses negligible class bias. This balanced performance underscores the model’s capacity to effectively delineate the subtle physiological boundaries between transient negative affect and pathological depression, thereby minimizing the risks of both false positives and false negatives in clinical screening.

To rigorously evaluate the proposed framework, we benchmarked it against SVM and RNN. While the SVM achieved a respectable accuracy of 86.3%, it still lagged behind the proposed method (92.2%), suggesting that traditional shallow classifiers are limited in capturing the complex non-linear interactions within multi-domain EEG features. It is worth noting that the RNN, which was included to investigate potential sequential dependencies in the spatial arrangement of channels. Its suboptimal accuracy (77.8%)—ranking lowest among the comparators—empirically indicates that multi-domain EEG features lack a linear sequential structure. Forcefully modeling them as a sequence appears to introduce noise rather than extract meaningful patterns. In contrast, the superior performance of our Multi-Head Additive Attention Network validates the effectiveness of processing feature subspaces in parallel. By adaptively weighing feature importance, our model successfully captures global discriminative patterns that both sequential and shallow baselines failed to fully exploit, the detailed quantitative comparison results are listed in Table 3.

While standard baselines provide a benchmark, recent deep learning advancements have shifted toward capturing subtle neural dynamics through complex architectures. Xia et al. [27] established the utility of multi-head self-attention for discerning MDD-related patterns, while Wei Liu et al. [28] underscored the necessity of temporal-spatial-frequency fusion. Yet, the performance gains in these models often come at the cost of substantial computational overhead and latency. Our framework reconciles this trade-off, achieving a competitive accuracy of 92.2% through a streamlined, lightweight design. Unlike the hybrid LSTM-SNN architecture by Sam et al. [29], which relies on computationally intensive sequential modeling, our attention-based fusion strategy efficiently captures global correlations without recurrent layers. This balance of precision and efficiency renders our approach uniquely suited for the resource-constrained environments of point-of-care screening.

## 4. Conclusions and Future Work

This study addresses the critical challenge of objective early depression recognition. We propose a robust classification framework that utilizes negative emotion as a physiological entry point to differentiate transient negative affective states from clinical depressive states. We constructed a comprehensive multi-domain feature set, identifying the spectral *α*/*β* PSD ratio, spatial *α*-asymmetry, and non-linear Sample Entropy as critical discriminators. Significance analysis confirmed that these features effectively capture the variations in signal complexity and dynamic regulation under different emotional states.

To synthesize these features, we proposed a novel neural network architecture incorporating a multi-head additive attention mechanism. This design allows the model to prioritize the most relevant feature subspaces across frequency, spatial, and non-linear domains, achieving a classification accuracy of 92.2% (F1-Score: 93%) and significantly outperforming baseline SVM and RNN models. Notably, compared to traditional deep learning models, our lightweight architecture demonstrates rapid convergence with fewer training iterations, providing a computationally efficient foundation for clinical diagnostic aids.

Despite the promising results, this study has limitations that direct our future research. First, we aim to enhance dataset diversity by incorporating larger cohorts across different ages and cultural backgrounds to validate model robustness against individual heterogeneity, a potential limitation of this study arises from the differing experimental paradigms of the two datasets: the HUSM dataset recorded resting-state EEG, while the DEAP dataset involved task-induced emotions. However, from a neurophysiological perspective, chronic depression is characterized by ‘trait-like’ neural signatures that persist regardless of immediate stimuli. In contrast, the negative emotions induced in healthy controls represent ‘state-dependent’ fluctuations. The network’s ability to distinguish these categories suggests it is capturing fundamental biomarkers of pathological rigidity versus healthy emotional flexibility. To ensure consistency, all signals were mapped to common channels within the international 10–20 system and subjected to unified baseline normalization. Second, beyond single-modality EEG, we plan to integrate multi-modal physiological signals and explore dynamic brain network connectivity to construct a more holistic model of emotional interaction. Ultimately, we intend to translate these findings into portable, low-cost monitoring devices, bridging the gap between algorithm development and practical mental health screening in daily life.

## Figures and Tables

**Table 1:** Feature significance analysis.

Channels	Fp1	Fp2	F3	F4	F7	F8	C3	C4
*p*-value	0.0042	0.0006	0.0081	0.0640	<0.0001	<0.0001	0.0005	0.0014

**Table 2:** Quantitative performance comparison of spatial asymmetry features using SVM and XGBoost classifiers.

Feature	Classifier	Accuracy	AUC
*α*-wave Asymmetry	SVM	73.14%	0.64
	XGBoost	91.14%	0.97
*β*-wave Asymmetry	SVM	70.49%	0.66
	XGBoost	89.22%	0.96

**Table 3:** The performance of the model was compared with other models.

Models	Accuracy	Precision	Recall	F1-Score
RNN	77.8%	86.9%	82.2%	84.5%
SVM	86.3%	86.95%	92.3%	89.5%
Ours	92.2%	93%	92%	93%

## Data Availability

The DEAP dataset analyzed during the current study is available in the Queen Mary University of London repository, http://www.eecs.qmul.ac.uk/mmv/datasets/deap/ (accessed on 10 July 2024). The HUSM dataset analyzed during the current study is publicly available in the Figshare repository, https://doi.org/10.6084/m9.figshare.4244171.v2 (accessed on 29 June 2024).
